# An Evaluation of Fluorouracil plus Paclitaxel and Oxiliplatin as a First-Line Treatment for Advanced Gastric Squamous Cell Carcinoma

**DOI:** 10.1155/2023/2176371

**Published:** 2023-04-06

**Authors:** Jie Qin, Yingpeng Shi, Xueli Zheng, Ya Lan, Mingxin Zhang, Mi Liu

**Affiliations:** ^1^Department of Gastroenterology, The First Affiliated Hospital of Xi'an Medical University, Xi'an 710077, Shaanxi, China; ^2^Department of General Practice, The First Affiliated Hospital of Xi'an Medical University, Xi'an 710077, Shaanxi, China; ^3^Cataract Refractive Center, Xianyang First People's Hospital, Xianyang 712000, Shaanxi, China; ^4^Department of Gastroenterology, Shangluo Central Hospital, Shangluo 726000, Shaanxi, China

## Abstract

**Objective:**

To investigate the efficacy of fluorouracil (FU) combined with paclitaxel (PTX) and oxaliplatin (OXA) as the first-line treatment for advanced gastric signet ring cell carcinoma (SRCC) and its influence on human epidermal growth factor receptor 2 (HER-2) expression.

**Methods:**

We collected one hundred and sixty-eight patients with advanced gastric SRCC, including 87 patients treated with FU combined with PTX and OXA as the study group (SG) and 81 patients treated with FU combined with OXA as the control group (CG). We compared indicators such as efficacy and adverse reactions after treatment between the two groups and also detected serum HER-2 expression pre- and post-treatment.

**Results:**

The incidence of adverse reactions differed insignificantly between SG and CG (*P* > 0.05). SG presented a notably higher objective response rate (ORR) and disease control rate (DCR) than that of CG (*P* < 0.05). After treatment, the serum HER-2 expression level of patients in both groups decreased significantly (*P* < 0.05), and that in SG was significantly declined compared to CG (*P* < 0.05). HER-2 was negatively correlated with the efficacy of both SG and CG. The 1-year survival rate in SG (29.89%) was significantly higher than that in CG (16.05%) (*P* < 0.05). The median OS and PFS were higher in DG than that in CG (*P* < 0.05).

**Conclusion:**

FU combined with PTX and OXA can effectively improve the efficacy of first-line treatment for advanced gastric SRCC while reducing HER-2 expression, without increasing the adverse reaction rate. This treatment is worthy of clinical promotion.

## 1. Introduction

Gastric cancer (GC) is the fifth most frequently diagnosed cancer worldwide and the third leading cause of cancer-related death [1, 2]. It is also the second-largest cancer-related death cause and the second most common invasive cancer in China, with approximately 500,000 people dying of GC in 2015 [3]. Although the overall incidence of GC has declined in recent decades, the incidence of gastric signet ring cell carcinoma (SRCC) is still increasing [4]. Gastric SRCC is mainly composed of scattered malignant cells containing cytoplasmic mucin, which accounts for more than 50% of the tumor [5], and is diagnosed as an adenocarcinoma based on the microscopic characteristics defined by the World Health Organization (WHO) [6]. Studies have shown that gastric SRCC and non-SRCC are considered to be unique biological entities originating from different carcinogens [7].

Most patients with gastric SRCC generally enter the hospital in the late stage due to nonspecific symptoms [8]. As the histological manifestation of gastric SRCC is characterized by poor adhesion and tends to invade through submucosal and subserosal pathways, the prognosis of advanced gastric SRCC is dreadful [9]. The treatment of patients with advanced gastric SRCC is similar to that of patients with other subtypes of GC [10], so it is necessary to develop and establish a variety of clinical treatment strategies to improve patient outcomes. Human epidermal growth factor receptor 2 (HER-2), a member of the HER family, is directly involved in the pathogenesis and progression of various human cancers [11, 12]. It is therefore often considered a bad prognostic factor [13, 14]. With the revolutionary influence of anti-HER-2 therapy in breast cancer patients [15], HER-2 and its blocking effect have been widely evaluated in other tumor types [16, 17]. The use of HER-2 inhibitor in GC has yielded favorable results and can be a prognostic factor in this disease [18]. FU is vital in the treatment of various cancers [19], and 5-FU has been identified as one of the standard first-line chemotherapy drugs for locally advanced or metastatic GC [20]. The combination of 5-FU and TRAIL had a greater inhibitory effect on the proliferation of gastric cancer cells than TRAIL alone. 5-FU significantly enhanced TRAIL-induced gastric cancer cell apoptosis. A new therapeutic strategy has been proposed to enhance the antitumor effect induced by 5-FU in GC cells resistant to 5-FU by TXN [21].

According to the Chicago consensus on peritoneal metastasis in 2020, PTX and OXA can be considered as the first-line chemotherapy for gastric cancer patients with peritoneal metastasis. In this study, we collected one hundred and sixty-eight patients with advanced gastric SRCC, including 87 patients treated with FU combined with PTX and OXA as the study group (SG) and 81 patients treated with FU combined with OXA as the control group (CG). Then, we tested indicators such as efficacy, adverse reactions, and HER-2 expression in two groups of patients under different treatment schemes to explore the therapeutic effect of fluorouracil (FU) combined with paclitaxel (PTX) and oxaliplatin (OXA) on advanced gastric SRCC and its influence on HER-2 expression.

## 2. Materials and Methods

### 2.1. General Information

One hundred and sixty-eight patients with advanced gastric SRCC in our hospital were collected as the research participants. Among them, 87 patients treated with FU combined with PTX and OXA were selected as the study group (SG), including 54 males and 33 females, with an average age of (69.37 ± 3.58) years. Eighty-one patients treated with FU combined with OXA were selected as the control group (CG), including 49 males and 32 females, with an average age of (70.28 ± 3.67) years.

### 2.2. Inclusion Criteria

Patients who were accompanied by family members and diagnosed with gastric SRCC by imaging and pathology were included, with TNM stage IIIb-IV, measurable lesions, Karnofsky performance status (KPS) score > 70, and complete clinicopathological data. All the patients had not received chemotherapy or other antitumor treatment within the last month, and the blood routine, liver and kidney function, and electrocardiogram were all normal before treatment.

### 2.3. Exclusion Criteria

Patients with an expected survival time of fewer than 3 months, previous history of mental illness and family history of mental illness, history of autoimmune deficiency, history of severe organ disease, and history of drug dependence were excluded. As well as those patients who cannot cooperate with the examination due to aphasia, irritability, confusion, and communication disorder. The experimental process was described to the patients and their families in advance, and this study was ratified by the Ethics Committee of our hospital, with the written informed consent obtained from the patients and their families.

### 2.4. Treatment of Patients

Patients in CG were given OXA 90 mg/m^2^ and 0.1 g calcium levofolinate intravenously for 3 h on the first day. On the second day, 0.1 g calcium levofolinate was given intravenously, followed by 48 h of intravenous infusion of FU 2000 mg/m^2^. One treatment cycle lasted for 15 days.

For patients in the SG, PTX 130 mg/m^2^ and 0.1 g leucovorin were given intravenously for 3 h on the first day, and dexamethasone was given twice at 5 mg/time, which was intravenously dripped 10 h and 30 min before PTX administration for pretreatment. On the second day, OXA (90 mg/m^2^) and folinate (0.1 g) were given intravenously for 3 h, followed by 48 h of intravenous infusion of FU 2000 mg/m^2^. The treatment took 15 days as a cycle.

Before chemotherapy, 5-HT3 receptor antagonists were routinely administered to prevent vomiting, and proton pump inhibitors were given to protect gastric mucosa. Patients should avoid exposure to ice-cold substances during medication. All patients received treatment for ≥2 cycles, and continued treatment for 2 cycles if the condition did not improve.

Before treatment and after two cycles of treatment, 5 ml of fasting venous blood was collected and loaded into an anticoagulant tube for 60 min (20–25°C). Thereafter, the samples were centrifuged at 1369.55xp and 4°C for 15 minutes with a centrifuge (Sichuan Shuke Instrument, Chengdu, China, TG 112), and then it was put into the −70°C cryogenic refrigerator for reserve. Serum HER-2 (Human HER-2 ELISA kit, Shanghai Yanhui biotechnology, PTGCN) level was detected by enzyme-linked immunosorbent assay (ELISA) strictly following the manufacturer's protocol.

### 2.5. Outcome Measures

The adverse reactions of two groups of patients during treatment were counted. After every 2 cycles of treatment, the therapeutic effects, which were divided into complete response (CR), partial response (PR), stable disease (SD), and progressive disease (PD), were recorded in the two groups. Objective response rate (ORR) = number of (CR + PR) cases/total number of cases, and disease control rate (DCR) = number of (CR + PR + SD) cases/total number of cases. Serum HER-2 levels were compared before and after 2 cycles of treatment, and the correlation between HER-2 and the curative effect of advanced gastric SRCC was analyzed. The patients were followed up for one year, and the 1-year survival rate, total survival time (OS, the time from drug use to death or the last follow-up), and progression-free survival time (PFS, the time from drug use to disease progression or death) of the two groups were compared statistically.

### 2.6. Statistical Methods

The results of this study were statistically analyzed by SPSS20.0 (IBM Corp, Armonk, NY, USA), and graphs were drawn with GraphPad Prism 7 (GraphPad Software Co., Ltd., San Diego, USA). Represented by *n*(%), the counting data between groups were compared by Chi-square test. The measured data were expressed by (*x* ± *s*), and the comparison between groups was made by *t*-test. Spearman's correlation coefficient was responsible for correlation analysis. *P* < 0.05 indicates that the difference was statistically significant.

## 3. Results

### 3.1. General Information

General information such as age, body mass index (BMI), smoking history, and drinking history of patients in the two groups were collected, as shown in Table 1. The average age in the study group was 69.37 ± 3.58 and 70.28 ± 3.67 in the control group. The BMI (kg/m^2^) in the study group and in the control group was 22.08 ± 2.04 and 22.24 ± 2.12, respectively. No significant difference was present in general information between SG and CG (*P* > 0.05).

### 3.2. Comparison of Adverse Reactions between the Two Groups

**Figure 1:**
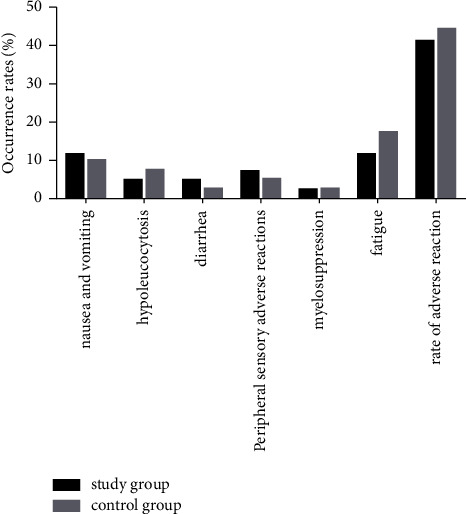
Comparison of adverse reactions between the two groups, with no significant difference.

See Figure 1 for the incidence of adverse reactions (fatigue, nausea and vomiting, leukopenia, etc.) between the two groups. The incidence of nausea and vomiting, diarrhea, and peripheral sensory adverse reactions were higher in study groups than in control groups. The myelosuppression and fatigue were lower in study groups than in control groups. There was no significant difference in the incidence of adverse reactions between SG (41.38%) and CG (44.44%) (*P* > 0.05), and both groups recovered after symptomatic treatment without chemotherapy-related death.

### 3.3. Comparison of Clinical Efficacy between the Two Groups

**Figure 2:**
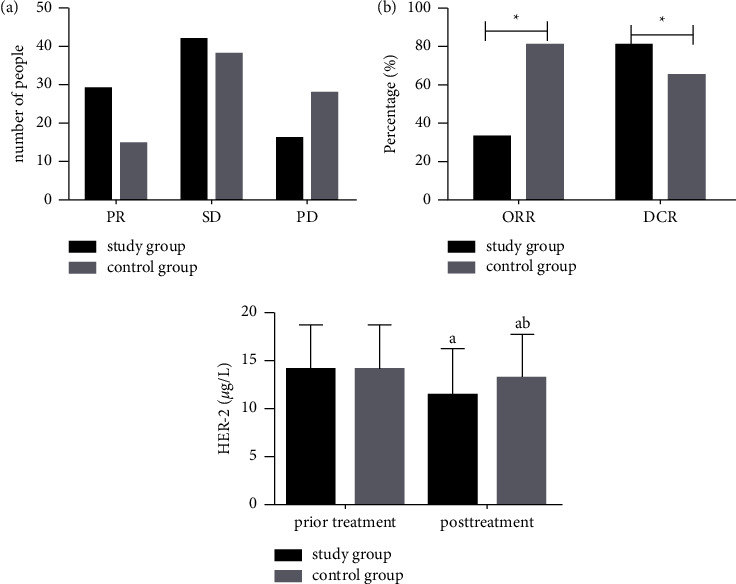
Comparison of serum HER-2 expression level between the two groups before and after treatment.

The curative effects of the two groups after treatment were compared, as shown in Figure 2. The number of people with PR and SD were higher than the control groups, while the SD was lower than in control groups. SG presented notably higher ORR and DCR (33.33%, 81.61%) than that of CG (18.52%, 65.43%) (*P* < 0.05).

### 3.4. Comparison of Serum HER-2 between the Two Groups Pre- and Post-Treatment

Serum HER-2 levels pre- and post-treatment were compared between the two groups, as shown in Figure 2. The serum HER-2 level did not differ remarkably between SG and CG before treatment (*P* > 0.05), but after treatment, it reduced evidently in both groups (*P* < 0.05), and the decrease was more significant in SG than in CG (*P* < 0.05).

There was no significant difference in HER-2 between the two groups before treatment, but it decreased significantly in both groups after treatment, and the HER-2 level was significantly lower in the study group than that in the control group.

Note: *a* indicates *P* < 0.05 compared within the same group before and after treatment; *b* indicates *P* < 0.05 compared with the study group after treatment.

### 3.5. Correlation between HER-2 and Curative Effect in the Two Groups

**Figure 3:**
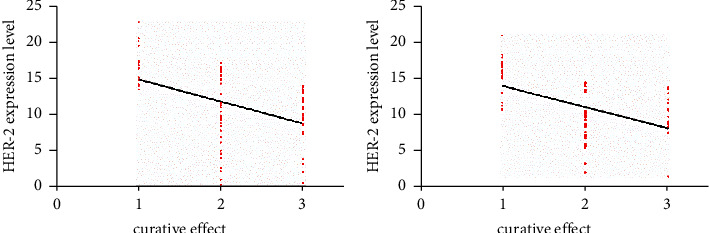
Correlation between HER-2 and curative effect of advanced gastric signet-ring cell carcinoma. (a) HER-2 was significantly negatively correlated with curative effect in the study group. (b) HER-2 was negatively correlated with the efficacy in the control group. Note: 1 indicates low PD, 2 indicates SD, and 3 indicates PR.

See Figure 3 for the correlation between HER-2 and the curative effect of advanced 0020030 gastric SRCC. HER-2 had a significant negative correlation with the curative effect in both SG and CG (*r* = −0.45, *r* = −0.52, *p* < 0.05).

### 3.6. Comparison of Survival between the Two Groups

**Figure 4:**
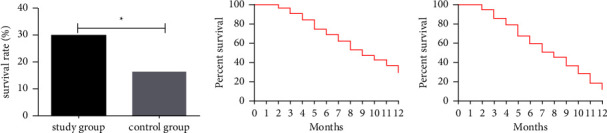
Comparison of 1-year survival rate between the two groups. (a) The 1-year survival rate of patients in the study group was significantly higher than that in the control group. (b) The 1-year overall survival rate of patients in the study group was 29.89%. (c) The 1-year overall survival rate of patients in the control group was 16.05%. Note: ^*∗*^indicates *P* < 0.05 compared between the two groups.

Statistics are made on the 1-year survival rate of patients in two groups, as shown in Figure 4. All the 168 patients were successfully followed up. The 1-year survival rate of SG (29.89%) was significantly higher than 16.05% of CG (*P* < 0.05).

### 3.7. Comparison of Median OS and PFS between the Two Groups

**Figure 5:**
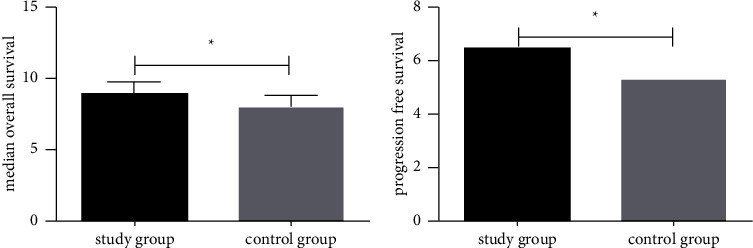
Comparison of median OS and PFS between the two groups. (a) The median OS in the study group was significantly higher than that in the control group. (b) The median PFS in the study group was significantly higher than that in the control group. Note: ^*∗*^indicates *P* < 0.05 compared between the two groups.

Median OS and PFS of the two groups were compared, as shown in Figure 5. The median OS and PFS in SG were higher compared to CG (*P* < 0.05).

## 4. Discussion

Gastric SRCC accounts for 4%–17% of all types of GC [22]. In the United States, gastric SRCC has an incidence of 0.094/100000 and a 5-year survival rate of 82.8%, and tumor stage and size are independent predictors of lymph node metastasis [23]. All the enrolled patients were followed up for 1 year, and the 1-year survival rate of patients was noticeably higher in SG than in CG, indicating that the treatment regimen used in SG can effectively improve the survival rate of patients with advanced gastric SRCC. Some reports have demonstrated that compared with the standard cisplatin plus FU regimen, PTX plus FU does not statistically prolong OS in patients with locally advanced esophageal squamous cell carcinoma [24]. Studies have shown that the survival rates of gastric SRCC in different periods are varying, and the five-year overall survival rates of early and late SRCC are 0.830 and 0.164, respectively [25]. Currently, chemotherapy is a primary means of clinical treatment for advanced GC [26].

FU is vital in the treatment of various cancers [19], and 5-FU has been identified as one of the standard first-line chemotherapy drugs for locally advanced or metastatic GC [20]. A new therapeutic strategy has been proposed to enhance the antitumor effect induced by 5-FU in GC cells resistant to 5-FU by TXN [21]. Some studies have also suggested that gastrectomy combined with OXA +5-FU has a definite therapeutic effect on GC, which can achieve a better short-term clinical therapeutic effect [27]. In Japan, oral FU plus cisplatin is the standard treatment for advanced GC, while PTX is an option. Clinically developed for breast cancer, nonsmall cell lung cancer and pancreatic cancer, PTX has also been clinically applied for the treatment of GC in Japan, and the results of the second-stage study have been published, indicating that this combination therapy is well tolerated and has yielded antitumor efficacy in patients with advanced GC [28], while OXA is one of the most extensively used chemotherapeutic agents in the treatment of various cancers including GC. However, due to its toxicity and drug resistance, its therapeutic indicator is narrow. It is necessary to develop new therapies to enhance efficacy and reduce toxicity [29]. Therefore, the purpose of this study was to explore the efficacy of FU combined with PTX and OXA in the treatment of advanced gastric SRCC.

Adverse reactions of chemotherapy mainly include gastrointestinal reactions, myelosuppression, and neurotoxic reactions. Implementation of family was advised to support the patients, and patients are supported by nutrition management intervention, which is helpful for patients to overcome the adverse reactions, such as nausea and vomiting [30]. Although there was no marked difference, the incidence of adverse reactions in the SG was still lower than that in the CG, which indicated that the treatment regimen applied in the SG could more effectively alleviate the pain and was more conducive to the successful completion of treatment for patients. Studies have shown that PTX combined with 5-FU is well tolerated and effective in the treatment of advanced GC [31]. Also, it is shown that chemotherapy based on FU and Kanglaite injection can enormously improve the clinical efficacy of patients with advanced gastrointestinal malignancies and reduce adverse reactions [32]. Therefore, this study further compared the efficacy of the two groups of patients after treatment, and found that the number of cases with ORR and DCR in SG was significantly higher than those in CG. A study [33] included 61 patients with gastric SRCC and found that there was 1 case of CR, 36 cases of PR, 15 cases of SD, and 9 cases of PD after treatment with docetaxel combined with cisplatin and FU, which was similar to this study, indicating that FU combined with PTX and OXA could profoundly improve the curative effect of advanced gastric SRCC. In the treatment of GC patients, the detection of HER2 expression has become a routine [34]. Human epidermal growth factor receptor 2 (HER-2), a member of the HER family, is directly involved in the pathogenesis and progression of various human cancers. It is therefore often considered as a bad prognostic factor. With the revolutionary influence of anti-HER-2 therapy in breast cancer patients, HER-2 and its blocking effect have been widely evaluated in other tumor types. The use of HER-2 inhibitor in GC has yielded favorable results and can be a prognostic factor in this disease. This study analyzed the correlation between HER-2 and the curative effect of advanced gastric SRCC, and concluded that HER-2 was significantly negatively correlated with the curative effect in both SG and CG, in other words, HER-2 increased with the decrease of the efficacy, indicating that HER2 could be used to judge the curative effect of advanced gastric SRCC. It was also found that serum HER-2 differed insignificantly between the two groups before treatment, but it decreased significantly in both groups after treatment, indicating that the two chemotherapy methods were effective for advanced gastric SRCC. Whereas, the HER-2 in SG was significantly lower than that in CG, suggesting that the treatment regimen of the SG could reduce HER-2 more effectively and improve the curative effect on advanced gastric SRCC. Gastric SRCC is mainly composed of scattered malignant cells containing cytoplasmic mucin, which accounts for more than 50% of the tumor, and is diagnosed as an adenocarcinoma based on the microscopic characteristics defined by the World Health Organization (WHO). Studies have shown that gastric SRCC and non-SRCC are considered to be unique biological entities originating from different carcinogens. Gastric cancer is one of the leading causes of cancer-related death worldwide. Many patients have inoperable disease at diagnosis or have recurrent disease after resection with curative intent. Literature has identified that the 5-year cumulative survival rate of gastric SRCC is 0.49, while the 5-year overall survival rate is 0.16, with 17.4% complications [25]. This is similar to the 1-year survival rate of patients in the CG in this study. Other reports have revealed that the addition of docetaxel to cisplatin and 5-FU regimen can profoundly improve the progression time and OS of untreated advanced GC patients with a median PFS of 7.2 months. Similar findings were obtained in the current study. The median OS and PFS of patients in SG treated with FU combined with PTX and OXA were higher than those in CG treated with OXA combined with 5-FU. This further indicated that the treatment plan used in SG can effectively improve the survival rate of patients with advanced gastric SRCC.

In this article, the curative effect, adverse reactions, and HER-2 of two groups of patients under different treatment schemes were tested to explore the curative effect of FU combined with PTX and OXA on advanced gastric SRCC and its influence on the expression level of HER-2, hoping to provide a theoretical basis for the treatment of advanced gastric SRCC. However, there are still some limitations in this study. The experimental subjects are limited, and the specific role of HER-2 in advanced gastric SRCC and the way in which the differential expression of HER-2 is caused are not clear. First-line treatment of FU combined with PTX and OXA can effectively improve the efficacy of first-line treatment for advanced gastric SRCC while enormously reducing HER-2 expression without increasing the adverse reaction rate [35].

## 5. Conclusion

Gastric cancer is one of the leading causes of cancer-related death worldwide. Many patients have inoperable disease at diagnosis or have recurrent disease after resection with curative intent. In this study, we tested the curative effect, adverse reactions, and HER-2 of two groups of patients under different treatment schemes to explore the curative effect of FU combined with PTX and OXA on advanced gastric SRCC and its influence on the expression level of HER-2. We hoped to provide a theoretical basis for the treatment of advanced gastric SRCC. However, there are still some limitations. The experimental subjects are limited, and the specific role of HER-2 in advanced gastric SRCC and the way in which the differential expression of HER-2 is caused are not clear. It is hoped that the research content will be improved continuously in the future research to provide more scientific reference for clinical treatment.

The novelty of this study was to show that FU combined with PTX and OXA can effectively improve the efficacy of first-line treatment for advanced gastric SRCC, without increasing the adverse reaction rate. This treatment is worthy of clinical promotion. However, there are also limitations of this study. The underlying mechanism was not so clear and which molecular that act with HER-2 was not clarified. Further studies are needed to study how HER-2 regulates the prognosis of gastric cancer.

## Figures and Tables

**Table 1:** Comparison of general data between the two groups (*x* ± *s*)/(*n*(%)).

	Study group (*n* = 87)	Control group (*n* = 81)	*t*/*X*^2^	*P*
Age (years old)	69.37 ± 3.58	70.28 ± 3.67	1.62	0.11
BMI (kg/m^2^)	22.08 ± 2.04	22.24 ± 2.12	0.50	0.62
Gender			0.04	0.83
Male	54 (62.07)	49 (60.49)	—	—
Female	33 (37.93)	32 (39.51)	—	—
Drinking history			0.00	0.99
Yes	59 (67.82)	55 (67.90)	—	—
No	28 (32.18)	26 (32.10)	—	—
Smoking history			0.07	0.79
Yes	53 (60.92)	51 (62.96)	—	—
No	34 (39.08)	30 (37.04)	—	—
III_b_	15 (17.24)	12 (14.81)	0.18	0.67
IV	72 (82.76)	69 (85.19)	—	—
Initial treatment	32 (36.78)	28 (34.57)	0.09	0.76
Retreatment	55 (63.22)	53 (65.43)	—	—

## Data Availability

The datasets used and/or analyzed during this study are available from the corresponding author on reasonable request.
